# Clinical risk factors for in-hospital mortality in older adults with HIV infection: findings from a South African hospital administrative dataset

**DOI:** 10.11604/pamj.2017.26.126.11000

**Published:** 2017-03-03

**Authors:** Kumeren Govender, Fatima Suleman, Yoshan Moodley

**Affiliations:** 1 Nelson R Mandela School of Medicine, University of KwaZulu-Natal, South Africa; 2 Discipline of Pharmaceutical Sciences, University of KwaZulu-Natal, South Africa; 3 Discipline of Anaesthesiology and Critical Care Medicine, Nelson R Mandela School of Medicine, University of KwaZulu-Natal, South Africa

**Keywords:** HIV, hospital mortality, risk factors, older adult

## Abstract

**Introduction:**

The proportion of older South African adults (aged ≥50 years old) with HIV infection requiring hospitalization is likely to increase in the near future. Clinical risk factors for in-hospital mortality (IHM) in these patients are not well described. We aimed to identify clinical risk factors associated with IHM and their overall contribution towards IHM in older South African adults with HIV infection.

**Methods:**

Clinical data for 690 older adults with HIV infection was obtained from the hospital administrative database at the Hlabisa Hospital in KwaZulu-Natal, South Africa. Logistic regression was used to determine independent clinical risk factors for IHM. Population-attributable fractions (PAFs) were calculated for all independent clinical risk factors identified.

**Results:**

Male gender (p=0.005), CD4 count <350 cells/mm^3^ (p=0.035), unknown CD4 count (p=0.048), tuberculosis (p=0.033) and renal failure (p=0.013) were independently associated with IHM. Male gender contributed the most to IHM (PAF=0.22), followed by unknown CD4 count (PAF=0.14), tuberculosis (PAF=0.12), renal failure (PAF=0.06) and CD4 count <350 cells/mm^3^ (PAF=0.01).

**Conclusion:**

Although further research is required to confirm our findings, there is potential for these clinical risk factors identified in our study to be used to stratify patient risk and reduce IHM in older adults with HIV infection.

## Introduction

Dramatic scale-up in the provision of antiretroviral therapy (ART) for HIV-infection in sub-Saharan Africa continues to improve the quality of life and radically reduce early mortality associated with HIV [1]. In sub-Saharan Africa it is estimated that ART coverage reached 41% in 2014, up from less than 1% in 2000 [2]. This has led to a substantial increase of the HIV prevalence in sub-Saharan Africa with nearly 11.5 million people initiated on therapy as of 2015 [2]. Mills et al. demonstrated that the life expectancy of those who are HIV-infected can approach the life expectancy of those who are not HIV-infected provided ART is initiated early (CD4 count >250 cells/µL) [3]. Therefore, as scale-up of ART in sub-Saharan Africa continues to rapidly expand, the life expectancy of the HIV-infected population continue to improve, resulting in a temporal transition of the HIV epidemic toward the older age groups. In Africa, the World Health Organization (WHO) defines older age as 50 years or more, due to new roles and loss of previous roles in society [4]. An increase in the prevalence of HIV in persons older than 50 years of age has already been reported in several developed countries. In the United States ART was first introduced in 1996, after which the prevalence of HIV in older adults (≥ 50 years old) grew from 17% in 2001 to 30% in 2008 [5, 6].

In Italy the proportion of older adults living with HIV increased from 4.9% in the years 1982-1990 to 15.9% in the years 2000-2005 [7]. Older adults in 2012 represented 9.5% (608 000 persons) of the total HIV burden in South Africa [8]. A study in rural South Africa quantified the aging of the HIV epidemic and predicted that the number of HIV-infected older adults would increase by 100% between 2004 and 2025 [9]. Moreover there is an increasing trend in the incidence of HIV in older adults reported in rural settings where health services are now becoming accessible [8]. Inevitably a proportion of these older adults with HIV infection will require hospitalization for various HIV-related or HIV-unrelated conditions during their lifetime [10–12]. Studies on HIV-infected individuals from the United States and Greece have shown higher comorbidities, longer lengths of admission and more frequent hospitalizations in older patients compared to younger patients [5, 13, 14]. Furthermore studies suggest higher inpatient mortality in HIV-infected older adult patients when compared to their HIV-infected younger counterparts [14]. In-hospital mortality (IHM) is associated with higher health care expenditure and is also an important indicator of a public health system´s response to the HIV epidemic [14, 15]. Considering the progressively high levels of IHM in older adults infected with HIV and its potential impact on resource utilization within public health systems, it is important that methods such as risk stratification systems be developed, that aim to reduce IHM in these patients. Risk stratification systems involve the identification of independent clinical risk factors within a population at risk of a poor outcome [16]. However, clinical risk factors that contribute to IHM in older adults infected with HIV are not well described, particularly in the South African context where the burden of HIV in this sub-population is amongst the highest in the world. We therefore sought to identify clinical risk factors associated with IHM and their overall contribution toward this poor outcome in older adults with HIV infection admitted to a South African hospital.

## Methods

**Study design and setting:** This study was a retrospective analysis of data collected at the Hlabisa Hospital between January 2011 and February 2015. The hospital provides services to over 230 000 people in the Hlabisa district, Mtubatuba and parts of the “Big Five” municipalities in the Northern KwaZulu-Natal Province, South Africa. The overall HIV incidence in the Hlabisa district has been estimated at 3.2 per 100 person years while the district prevalence of HIV in the older adult is estimated at 9.5% and predicted to further rise [8]. Other characteristics of the Hlabisa population are described elsewhere [17].

**Data source and definitions:** HIV-positive patients aged ≥ 50 years old who were admitted to the Hlabisa Hospital between January 2011 and February 2015 were identified through review of hospital administrative data collected as part of the Africa Centre Demographic Information System (ACDIS) [17]. Patients were considered HIV-infected if they had an HIV-positive serostatus recorded in the database. Demographic characteristics analysed included gender and age; and clinical characteristics analysed included HIV status, ART status, CD4 count, and co-morbidity. Patient comorbidities were identified from the discharge diagnoses fields listed in the administrative database using the : *International Statistical Classification of Diseases and Related Health Problems-Tenth revision* (ICD-10) code groupings proposed by the American Healthcare Cost and Utilization Project (HCUP) [18]. The most prevalent co-morbidities were included in the final statistical analysis in accordance with other similar studies on IHM [19].

**Statistical analysis:** Univariate and multivariate statistical testing of the final dataset was undertaken. All statistical analyses were performed using the Statistical Package for the Social Sciences (SPSS) version 23 (IBM Corp., Armonk, NY, USA).

**Univariate statistical methods:** Gender, ART status, CD4 count, and co-morbidities were converted to categorical variables. Categorical data was analysed using χ^2^ tests or Fishers Exact test, where appropriate. Age data was treated as a continuous variable. When tested for normality using the Shapiro-Wilk test, age data was not found to be normally distributed and was therefore analysed using a Mann-Whitney U test. Results of the univariate statistical testing are presented as frequencies and percentages, or medians and interquartile ranges (IQRs), where appropriate.

**Multivariate statistical methods:** Logistic regression was used to identify independent associations between clinical characteristics and IHM. Clinical characteristics with p<0.2 from the univariate testing were included in the logistic regression analysis. This purposeful selection of clinical characteristics ensured that we did not potentially violate the “10 outcome events per single variable” rule of thumb for logistic regression analyses and also ensured that we would obtain a parsimonious regression model [20, 21]. A Hosmer-Lemeshow test was used to measure model fit. Results were presented as odds ratios (OR) with a confidence interval of 95% (CIs). Results with a p-value <0.05 were considered statistically significant.

**Population-attributable fraction (PAF):** The PAF is defined as the proportion of cases in the population that would not have occurred in the absence of the exposure [22]. In this study the PAF was calculated for each independent risk factor identified from the binary logistic regression analysis (clinical characteristics with p<0.05 in the regression model) using conventional methods [22].

**Ethical approval:** Ethical approval for this study was obtained from the University of KwaZulu-Natal Biomedical Research Ethics Committee (Protocol EXM277/15 & Protocol BE281/16).

## Results

**Figure 1:**
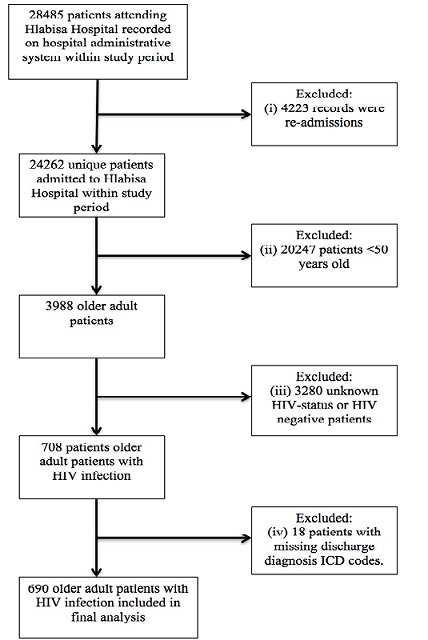
Study profile

Following application of the relevant inclusion and exclusion criteria (Table 1), the final study population consisted of 690 older adult patients with HIV infection admitted to the Hlabisa Hospital between January 2011 and February 2015 (Figure 1). The cumulative incidence of IHM in the study population was 27.1% (95% CI: 23.9-30.5%). The baseline clinical characteristics of the entire study population are shown in Table 2. The median age of the entire study population was 58.0 (IQR: 52.0-61.0) years old. Approximately 51.2% of the entire study population was male. Of the entire study population, 45.4% were receiving ART, 53% were not receiving ART, and ART status could not be established in 1.6% of patients. Nine percent of patients in the entire study population had known CD4 counts <350 cells/mm^3^, 4.1% had CD4 counts ≥350 cells/mm^3^, while 87% of the entire study population did not have CD4 counts recorded. The ten most prevalent comorbidities in the entire study population were: Tuberculosis, acute bronchitis, acute and unspecified renal failure, intestinal infection, essential hypertension, anaemia, pneumonia (excluding that caused by tuberculosis), diabetes mellitus without complications, mycoses, and meningitis (excluding TB meningitis).

**Table 1:** Inclusion and exclusion criteria used to derive the study population

Criteria number	Inclusion criteria	Exclusion criteria
i	First admission to hospital during the study period	Subsequent admissions for patients who are already included in the study
ii	Age ≥ 50 years old	Age <50 years old
iii	HIV-positive patients	Unknown HIV-status or HIV-negative
iv	Patients with valid discharge ICD-10 codes	Patients with invalid or missing discharge ICD-10 codes

**Table 2:** Clinical characteristics of the study population expressed as a frequency (%)

Clinical Characteristic	Total Cohort (N=690)	Patients without in-hospital mortality (N=503)	Patients with in-hospital mortality (N=187)	p-value
Median age in years (Interquartile range)	58.0 (52.0-61.0)	55.0 (52.0-61.0)	56.0 (52.0-61.0)	0.291
Male gender	353.0 (51.2)	237.0 (47.1)	116.0 (62.0)	0.001
ART status				0.493
On treatment	313.0 (45.4)	227.0 (45.1)	86.0 (46.0)	
Not on treatment	366.0 (53.0)	266.0 (52.9)	100.0 (53.5)	
Unknown	11.0 (1.6)	10.0 (2.0)	1.0 (0.5)	
CD4 count				0.045
<350 cells/mm^3^	62.0 (9.0)	43.0 (8.5)	19.0 (10.2)	
>350 cells/mm^3^	28.0 (4.1)	26.0 (5.2)	2.0 (1.1)	
Unknown	600.0 (87.0)	434.0 (86.3)	166.0 (88.8)	
Tuberculosis	263.0 (38.1)	178.0 (35.4)	85.0 (45.5)	0.017
Acute Bronchitis	80.0 (11.6)	62.0 (12.3)	18.0 (9.6)	0.325
Acute and unspecified renal failure	77.0 (11.2)	47.0 (9.3)	30.0 (16)	0.013
Intestinal infection	73.0 (10.6)	54.0 (10.7)	19.0 (10.2)	0.827
Essential hypertension	65.0 (9.4)	55.0 (10.9)	10.0 (5.3)	0.026
Anaemia	45.0 (6.5)	32.0 (6.4)	13.0 (7.0)	0.780
Pneumonia (excluding that caused by tuberculosis)	42.0 (6.1)	27.0 (5.4)	15.0 (8.0)	0.195
Diabetes Mellitus without complications	36.0 (5.2)	29.0 (5.8)	7.0 (3.7)	0.288
Mycoses	32.0 (4.6)	19.0 (3.8)	13.0 (7.0)	0.078
Meningitis (excluding TB meningitis)	25.0 (3.6)	17.0 (3.4)	8.0 (4.3)	0.575
Surgery with general anaesthesia	2.0 (0.3)	2.0 (0.4)	0.0 (0.0)	0.388

Univariate statistical associations between patient clinical characteristics and IHM are also reported (Table 2). Male gender (p=0.001), CD4 cell count (p=0.045), tuberculosis (p=0.017), renal failure (p=0.013) and essential hypertension (p=0.026) met the criteria for inclusion in the multivariate analysis (p<0.2 from the univariate analysis). Five factors (Male gender (p=0.005), CD4 count <350 cells/mm^3^, (p=0.035), unknown CD4 count (p=0.048), tuberculosis (p=0.033) and renal failure (p=0.013)) were found to be independently associated with a higher risk of IHM in the logistic regression analysis (Table 3). When entered into the logistic regression analysis, variables such as essential hypertension, pneumonia (excluding that caused by TB) and mycosis were not independently associated with an increased risk of IHM (Table 3). The Hosmer-Lemeshow test indicated adequate regression model fit (p>0.05). The PAF obtained for the independent risk factors obtained from the logistic regression analysis (Table 4) suggested male gender to have the highest contribution to IHM (PAF=0.22) followed by unknown CD4 cell count (PAF=0.14), tuberculosis (PAF=0.12), renal failure (PAF=0.06) and CD4 cell count <350 cells/mm^3^ (PAF=0.01).

**Table 3:** Results of multivariate analysis: clinical characteristics independently/not independently associated with in-hospital mortality

Clinical Characteristic#	OR (95%CI)	p-value
Male gender	1.67 (1.17-2.38)	0.005
CD4 count <350 cells/mm^3^+	5.29 (1.13-24.85)	0.035
CD4 count unknown+	2.23 (1.01-4.95)	0.048
Tuberculosis	1.47 (1.03-2.10)	0.033
Acute and unspecified renal failure	1.91 (1.15-3.19)	0.013
Essential hypertension	0.54 (0.26-1.10)	0.087
Pneumonia (excluding that caused by TB)	1.35 (0.69-2.64)	0.389
Mycoses	1.81 (0.85-3.84)	0.122

# Variables including age, ART status, acute bronchitis, intestinal infection, anaemia, diabetes mellitus without complications, meningitis (excluding TB meningitis) and surgery with general anaesthesia did not reach statistical significance of p<0.2 from the univariate analysis and were not included in the multivariate analysis.

+ When compared to a CD4 ≥350 cells/mm^3^ reference group.

**Table 4:** Population-attributable fractions (PAFs) for clinical variables independently associated with in-hospital mortality

Clinical Characteristic	PAF
Male gender	0.22
CD4 count unknown	0.14
CD4 count <350 cells/mm^3^	0.01
Tuberculosis	0.12
Acute and unspecified renal failure	0.06

## Discussion

Almost 1 in 4 older adults (27 in every 100 older adult patients) with HIV infection suffered IHM in our study. This estimate is far higher than that described in similar populations from developed world countries such as the USA [14], but still lies within the range of estimates reported in a study from another African country, Malawi (20-30 deaths per every 100 patients) [19]. Independent associations between several clinical characteristics (Male gender, CD4 count, tuberculosis, and renal failure) and a higher risk of IHM in older adults with HIV infection were noted in our study. Male gender was associated with a 67% higher risk of IHM when compared with female gender, and accounted for almost 1 in 5 patient deaths. Male gender is associated with delayed presentation to healthcare facilities [23], and this may have been the case in our study where far more patients who were male presented with advanced HIV disease and subsequently suffered poorer outcomes when compared with their female counterparts. CD4 count measurements are important for determining staging of HIV disease and were required to determine a patient's eligibility for antiretroviral therapy. Our findings with regard to a low CD4 count are in keeping with the findings of a systematic review by Wagjanga et al., which also identified low CD4 count measurements, a proxy for late presentation for care, as an important determinant of inpatient survival [24]. Our findings for patients with an unknown CD4 count suggest that a large proportion of these patients might potentially have low CD4 counts, and opportunities for intervention in these patients are missed. The PAFs obtained for the CD4 count categories suggests that a known low CD4 count contributes less to overall mortality than an unknown CD4 count measurement, which is likely due to known immunocompromised patients receiving subsequent treatment. These findings further highlight the importance of obtaining CD4 count measurements in patients with HIV infection.

HIV infection substantially increases the risk of tuberculosis, a concerning relationship considering the high burden of tuberculosis in the general South African population. UNAIDS reports that tuberculosis is among the leading causes of mortality in people living with HIV infection [25]. This is supported by a South African autopsy study by Cox et al., which found that a large proportion of deaths in patients with HIV infection were likely caused by tuberculosis [26]. Our findings suggest a 47% higher risk of IHM in HIV and tuberculosis co-infected patients, and do not seem out of place considering the high burden of tuberculosis and HIV in the South African setting, with 12 in every 100 inpatient deaths in older adults with HIV infection being attributed to tuberculosis. Active case finding and adequate treatment of tuberculosis among the HIV-infected population remains a key intervention for reducing morbidity and mortality in our setting. Persons with HIV may develop renal failure as a result of the nephrotoxic side effects of some antiretroviral therapy drugs, or through nephritis resulting from the direct infection of the kidney by HIV or opportunistic infections. Acute renal failure is estimated to occur in up to 30% of patients with HIV infection, and is much more common in this patient group when compared to HIV-uninfected patients [15, 27]. The implications of renal failure on subsequent patient outcomes are marked, often linked with higher morbidity and mortality in afflicted patients [28–30]. This might explain our findings for a 91% higher risk of IHM in patients with renal failure, with 6 in every 100 inpatient deaths in our study being attributed to renal failure.

Our study did not identify independent associations between non-communicable comorbid disease (besides renal failure) and IHM. This finding may be related to infectious diseases contributing to mortality in a rural HIV-infected population rather than non-communicable diseases. This is further supported by the finding of communicable conditions comprising the majority of the most common patient comorbidities in our study. However, it is important to note that South Africa has begun a state of epidemiological transition, whereby higher disease burden appears to be shifting away from communicable diseases toward non-communicable diseases [31, 32]. It is likely that in the future non-communicable comorbid diseases will become more important in the context of patient outcomes in South Africa. Increasing age above 50 years old was also not associated with a higher risk of IHM in this study. Increasing age is usually associated with increased non-communicable disease comorbidity [33], which potentially increases the risk of future poor patient outcomes. However, we report a low burden of communicable disease in this study, despite the age of our study population. This might explain the observed lack of association between age and IHM in our study. No independent associations (either protective or harmful) with IHM could be established for anaemia and antiretroviral therapy status in our study. It is possible that anaemia was under-diagnosed, as anaemia is usually described as a common haematological abnormality in patients with HIV infection which is associated with poor survival [34]. In the general HIV-infected population, antiretroviral therapy use is associated with a reduction in mortality with HIV-infected individuals on ART now achieving near-normal life expectancies [3, 35]. Notably, almost half our study population was receiving antiretroviral therapy, which might explain our findings for this characteristic.

**Study limitations:** Data related to a number of potentially relevant patient clinical characteristics, for example body mass index, were not collected as part of the hospital administrative database. Furthermore, data related to medication use (other than antiretroviral therapy) and laboratory results (other than CD4 count measurements) were not collected. As such we were unable to investigate the impact of these clinical characteristics on the incidence of IHM. The specific pathological cause of death was indeterminable from the hospital administrative database, and we therefore describe all-cause mortality in this study. Future studies employing a prospective study design with an emphasis on collecting data on potentially relevant population-specific characteristics are required to overcome the aforementioned study limitations. There were also several limitations identified which can only be overcome through health system strengthening. For instance, this study limited its analysis to inpatient outcomes as patient survival status following hospital discharge could not be reliably ascertained. Patient tracking and use of community caregivers to conduct home visits might improve follow-up of discharge patients [36]. In addition, a large proportion of the CD4 count measurement data was missing. The challenge of missing CD4 count measurements has been identified elsewhere [37], and it once again represents a challenge which can only be overcome through health systems approaches such as the rollout of point of care CD4 count machines and the development of relevant checklists to improve CD4 count measurement recording [38]. The data used in our study was collected from a single, rural hospital and the findings of this study may not be generalizable to other South African hospital settings. Multisite studies, involving several institutions located in both rural and urban settings are required. While our study does have limitations, it still provides an important account of clinical risk factors for IHM in older patients with HIV infection, which can be used to develop future interventions aimed at reducing patient risk.

## Conclusion

Almost 1 in 4 older adult patients with HIV infection in our study suffered IHM. Male gender, a CD4 count <350 cells/mm^3^, an unknown CD4 count, tuberculosis and renal failure were important predictors of a higher risk of IHM in our study. The proportion of IHM attributed to each of these clinical risk factors ranged from 0.01 for CD4 count <350 cells/mm^3^, to 0.22 for male gender. While the most prevalent non-communicable diseases investigated in this study did not appear to contribute to IHM when compared with communicable disease (with the exception of renal failure), it is likely that this situation will change in the near future. Further research is required not only to validate the findings of our study, but to also overcome limitations identified in our study. It is hoped that our findings will be used to develop interventions aimed at reducing the risk of IHM in older adults, such as risk stratification systems.

### What is known about this topic

- The proportion of older South African adults (aged ≥ 50 years old) with HIV infection requiring hospitalization is likely to increase in the near future;
- Clinical risk factors for in-hospital mortality (IHM) in these patients are not well described.

### What this study adds

- This study adds to the body of evidence that almost 1 in 4 older adults (27 in every 100 older adult patients) with HIV infection suffered IHM in our setting;
- Male gender, a CD4 count <350 cells/mm^3^, an unknown CD4 count, tuberculosis and renal failure were important predictors of a higher risk of IHM in older HIV-infected adult patients;
- The study hopes that our findings will be used to develop interventions aimed at reducing the risk of IHM in older adults, such as risk stratification systems.
